# Bone metabolism and inflammatory characteristics in 14 cases of chronic nonbacterial osteomyelitis

**DOI:** 10.1186/s12969-017-0183-z

**Published:** 2017-07-11

**Authors:** Yurika Ata, Yutaka Inaba, Hyonmin Choe, Naomi Kobayashi, Jiro Machida, Naoyuki Nakamura, Tomoyuki Saito

**Affiliations:** 1 Department of Orthopaedic Surgery, Yokohama City University, 3-9 Fukuura, Kanazawa-ku, Yokohama, Japan; 2 Department of Orthopaedic Surgery, Kanagawa Children’s Medical Center, 2-138-4 Mutsukawa, Minami-ku, Yokohama, Japan

**Keywords:** Chronic nonbacterial osteomyelitis, Bone metabolism, Magnetic resonance imaging, Fluorodeoxyglucose-PET, Inflammatory biomarker

## Abstract

**Background:**

Chronic nonbacterial osteomyelitis (CNO) is a multifocal autoinflammatory disease that often impairs daily life in children. This study aimed to investigate the bone metabolic and inflammatory characteristics of patients with CNO, and to assess the differences between responders and nonresponders to conservative treatment.

**Methods:**

We investigated the clinical symptoms; laboratory data including inflammatory and bone metabolic biomarkers; and imaging findings from plain radiography, magnetic resonance imaging (MRI), fluorodeoxyglucose-positron emission tomography (FDG-PET), and dual-energy x-ray absorption (DEXA) in 14 patients with CNO. All patients underwent first-line treatment comprising systemic nonsteroidal anti-inflammatory drugs with or without bisphosphonate. According to the response to the first-line treatment, the patients were divided into the clinical remission/partial response group and the no response group. The differences in bone metabolic and inflammatory characteristics between the two groups were assessed.

**Results:**

All patients had low bone mineral density assessed with DEXA. The bone metabolic biomarkers (bone-specific alkaline phosphatase and tartrate-resistant acid phosphatase 5b) were increased in boys of all ages and in young girls. Multiple inflammatory regions were detected in all patients by using FDG-PET including asymptomatic regions. The no response group had higher immunoglobulin G (IgG) and a greater number of bone inflammatory lesions detected on MRI than the clinical remission/partial response group.

**Conclusion:**

Our data indicate the involvement of abnormal bone turnover, necessity of whole-body scanning, and association of higher serum IgG levels and greater numbers of inflammatory lesions with prolonged disease activity in patients with CNO.

## Background

Chronic nonbacterial osteomyelitis (CNO) is a pediatric autoinflammatory disease characterized by multifocal bone inflammation that predominantly involves metaphysis of bones without microbiological osteomyelitis [1, 2]. It is also known as chronic recurrent multifocal osteomyelitis (CRMO) [3–8]. Diagnosis can be made on the basis of clinical signs, laboratory data, microbial and histopathological analyses, and radiological findings. Because of the varied clinical features, the establishment of universal diagnostic criteria and guidelines for the treatment of this disease remains challenging [6, 9–11].

Imaging is an important diagnostic tool for the diagnosis and detection of inflammatory lesions. Plain radiography may reveal sclerotic, lytic, or mixed lesions; however, these are not specific for CNO [12]. Magnetic resonance imaging (MRI) has been demonstrated to be useful in scanning affected bone lesions [2, 11, 13]. Technetium-99 bone scan permits the recognition of multiple unsuspected osseous lesions [14, 15]. Fluorodeoxyglucose-positron emission tomography (FDG-PET) can be used for whole-body scanning and for quantification of the severity of inflammation through the measurement of the standardized uptake value (SUV) [16]; however, the utility of FDG-PET in CNO has not been well investigated previously.

First-line therapy for CNO is nonsteroidal anti-inflammatory drugs (NSAIDs) [11]. Bisphosphonates are used in combination with NSAIDs in several previous reports [17, 18]. Regardless of the first-line therapy, severe pain still disrupts the daily life of some patients [19]. In cases with no response to the first-line therapy, biological therapy or disease-modifying antirheumatic drugs (DMARDs) may be effective [20–22]. Although biological therapy or DMARDs may have adverse effects, previous study demonstrated advantage of aggressive therapy from the initiation of treatment [23]. Therefore, prediction of nonrespose to first-line therapy may enable avoiding the long-term impairment of daily life in children with intractable CNO. However, only a few studies have focused on the characteristics of patients who were not responsive to conservative treatment for CNO [24].

This study aimed to assess the demographic, inflammatory, and bone metabolic characteristics of patients with CNO, and to investigate the characteristics of patients not responding to the first-line treatment of NSAIDs alone or NSAIDs in combination with a bisphosphonate. For this purpose, we performed serological assessment of systemic inflammation and bone metabolism, and utilized imaging modalities including FDG-PET and MRI to visualize local inflammation in bones and exclude other diseases, as well as dual-energy x-ray absorption (DEXA) to quantify bone metabolism.

## Methods

### Patients

This study was approved by our institutional review board. Patients younger than 18 years who were suspected of having CNO at our institutions (Yokohama City University Hospital and Kanagawa Children’s Medical Center) between 2011 and 2014 were screened for clinical symptoms, serum laboratory data, radiological findings including FDG-PET and focal MRI, and bone biopsy followed by microbiological culture and histological analysis after they provided informed consent. The diagnosis of CNO in the current study was made if the following criteria were all fulfilled: (i) clinical findings of regional pain with or without localized swelling existing for >2 months; (ii) regional inflammation in bones detected on imaging (increased multifocal uptake in FDG-PET or FDG-PET/CT, or markedly high signal intensity on T2-weighted short tau inversion recovery [T2-STIR] MRI); and (iii) negative findings for infections, benign or malignant tumors, and other disorders on tissue examination.

By using the diagnostic criteria, we enrolled 14 children (5 girls, 9 boys) with a diagnosis of CNO in this study. There was no patient who refused to participate in our study. The collected data included age at diagnosis, sex, time required for diagnosis after the initial symptoms, follow-up duration, clinical presentation, imaging findings, and serum laboratory data.

### Imaging assessments

Imaging assessments consisted of FDG-PET, MRI, and DEXA. FDG-PET (SET 2400 W; Shimadzu, Kyoto, Japan) was conducted between 2011 and 2014. From 2014, FDG-PET/CT (SET 2400 W, Shimadzu) was conducted on patients. PET images were obtained with 59.5- and 20.0-cm transverse fields of view, which produced 63 image planes with a 3.125-mm interval between images. The transverse resolution at the center of the field of view was 4.2 and 5.0 mm full width at half-maximum. Measurements with FDG-PET and FDG-PET/CT included the number and location of high-uptake regions and the maximum SUV (SUV max) in affected regions. Organs throughout the body were also carefully examined for the possibility of benign or malignant tumors. The locations of clinical symptoms or multifocal uptake regions detected on FDG-PET were thereafter investigated by using MRI for the focal assessment of bone abnormalities. MRI was performed with a 1.5-T system (Symphony; Siemens, Erlangen, Germany) with a body matrix phased-array coil. Each patient was placed in the supine position, and the MRI protocol included standard sequences. The MRI findings included the numbers and locations of bone with markedly high signal on T2-STIR. DEXA (QDR 2000; Hologic, Waltham, MA, USA) was used to assess the bone mineral density (BMD) of the second to fourth lumbar spines in frontal view. BMD was converted to age- and sex-matched Z-scores by using previously reported average BMD for each age [25].

### Serum laboratory data

Serum laboratory data included C-reactive protein (CRP), erythrocyte sedimentation rate (ESR) for the assessment of systemic inflammation, serum amyloid A (SAA), immunoglobulin G (IgG) for the determination of immunological reaction, bone alkaline phosphatase (BAP) as the biomarker for bone formation, and tartrate-resistant acid phosphatase serum band 5b (TRAP5b) as the biomarker for bone resorption. Because boys and girls have different trends in bone metabolism, the BAP and TRAP5b values of boys and girls with CNO were normalized by using previously reported age- and sex-matched mean data [25]. The BAP and TRAP5b values of boys and girls with CNO were independently plotted to visualize the abnormality in the chart with the previously reported normal range at each age for BAP and TRAP5b (−1.88SD to +1.88SD from the normal mean) [25].

### Treatment

All patients were treated with NSAIDs as first-line therapy but patients whose symptoms failed to be controlled with NSAIDs alone were treated in combination with a bisphosphonate (alendronate, 35 mg/week). Response to treatment was determined by assessing the improvement of local pain and MRI findings at 6 months after the initiation of first-line therapy. Improvement of MRI findings was assessed by comparing the numbers of inflammatory lesions before and after treatment. The treatment effect was evaluated as “no response” (no change in pain and numbers of inflammatory lesions on MRI), “partial response” (decrease in pain and/or numbers of inflammatory lesions on MRI), or “clinical remission” (disappearance of inflammatory lesions on MRI and clinical symptoms including pain, redness, swelling, or heat sensation). For patients with no response, tumor necrosis factor (TNF)-α antibody and methotrexate treatment were started as a second-line therapy.

### Statistical analysis

We assigned patients with partial response and clinical remission into group 1, and patients with no response into group 2. For the statistical analysis of the data, JMP 11 statistical software (SAS Institute Inc., Cary, NC, USA) was used. The differences of demographic data, serum laboratory data, and imaging findings between groups 1 and 2 were assessed by using chi-square test and Student’s t-test or Mann-Whitney U-test in data sets that were not normally distributed. A *p*-value of <0.05 was considered statistically significant.

## Results

The 14 patients with CNO consisted of 5 girls and 9 boys with a mean age of 11 years (range, 8–15 years) at the first symptom. The mean time required for diagnosis after the first symptom was 8 months (range, 2–16 months), and the patients had a mean serum CRP of 0.6 mg/dL (range, 0.01–3.49 mg/dL), mean ESR of 23 mm/h (range, 1–77 mm/h), mean SAA of 48 μg/mL (range, 5–143 μg/mL), and mean IgG of 1288 mg/dL (range, 802–1722 mg/dL) at the first presentation. These patients were followed for a mean of 39 months at our institutions (Table 1).

**Table 1:** Clinical, laboratory, and demographic features of patients with chronic nonbacterial osteomyelitis

	Chief compliment	Sex	Age (years)	The time between first symptom and diagnosis(months)	Follow-up (months)	CRP (mg/dL)	ESR (mm/h)	SAA (μg/mL)	IgG (mg/dL)	BAP (μg/L)	TRAP5b (mU/dL)	Number of bones with high accumulation on FDG-PET	Bone lesions with high signal on T2-STIR MRI	BMD(g/cm^2^)	Z score	Treatment	Treatment response to first line therapy
1	Fever of unknown origin	F	13	18	46	0.09	33	27	1722	37.4	338	8	33	0.921	−0.8	N + B	no response
2	Pain in the left hand	M	15	3	44	0.01	5	<5	1620	42.3	1870	8	24	0.726	−3.7	N	no response
3	Pain in the right foot	M	12	12	45	0.49	35	56	1524	82.8	1170	1	29	N/A	N/A	N	no response
4	Pain in both femurs	M	12	4	56	2.56	59	143	1561	53.5	1050	8	8	0.582	−2.1	N + B	no response
5	Pain in both hands	F	11	9	26	0.01	2	<5	802	30.2	239	2	10	0.667	−2.2	N + B	clinical remission
6	Pain in the right foot	M	12	5	48	0.01	1	<5	1057	62.8	1620	3	3	0.742	−1.5	N + B	clinical remission
7	Fever of unknown origin	F	15	3	52	0.4	18	26	1361	8.1	270	11	27	0.999	−0.3	N + B	partial response
8	Pain in both knees	F	5	2	33	0.02	7	<5	993	65.5	914	4	8	N/A	N/A	N + B	clinical remission
9	Fever of unknown origin and pain in the left knee	M	10	24	22	0.54	77	58	1561	49.49	2150	2	2	0.601	−1.4	N	partial response
10	Pain in both feet and knees	M	13	13	31	0.04	5	<5	1255	116.4	2430	4	4	0.688	−2.1	N + B	partial response
11	Pain in both feet	F	10	11	31	0.01	4	<5	1255	62.2	843	4	4	0.593	−2.8	N + B	clinical remission
12	Pain in both feet	M	12	4	32	0.19	16	14	1150	94.2	1500	5	2	0.505	−2.9	N + B	partial response
13	Pain in the right foot	M	8	5	29	0.01	2	6	1293	64.1	717	1	3	0.554	−1.9	N + B	partial response
14	Pain in both feet and knees	M	10	3	22	3.49	51	50	879	72	1320	6	8	0.513	−2.6	N + B	partial response
Mean		Girls: 5 Boys: 9	11.3	8.3	37	0.6	23	48	1288	60	1174	4.8	11.8	0.7	−2.0	NSAIDs alone: 3 NSAIDs + Bisphosphonate: 11	Clinical remission: 4 Partial response: 6 No response: 4

*Abbreviations*: *F* female, *M* male, *CRP* C-reactive protein, *ESR* erythrocyte sedimentation rate, *SAA* serum amyloid A, *IgG* immunoglobulin G, *BAP* bone alkaline phosphatase, *TRAP5b* tartrate-resistant acid phosphatase serum band 5bm, *BMD* bone mineral density﻿, *N*NSAIDs, *B* Bisphosphonate

Twelve cases (86%) showed multifocal inflammatory regions on FDG-PET (Table 1). FDG-PET detected the locations of clinical symptoms as inflammatory foci with high accumulation of FDG in all cases. FDG-PET detected a mean of 4.9 high-uptake regions/case, and the mean value of SUV max was 1.84 (range, 0.9–4.1). Three of 14 patients had a high-uptake region in the vertebral body, although none of them complained of back pain. No patients were suspected of having a benign or malignant tumor in organs. MRI showed a markedly high signal in a mean of 11.8 bones/case on T2-STIR.

**Fig. 1:**
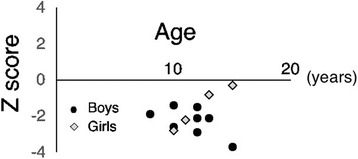
BMD of the lumbar spine expressed as Z-scores in patients with CNO. The BMD of the lumbar spine in all patients was lower than the age-matched normal BMD (not available in two patients). The mean Z-score at the lumbar spine in our patients with CNO was −1.7. BMD, bone mineral density; CNO, chronic nonbacterial osteomyelitis

**Fig. 2:**
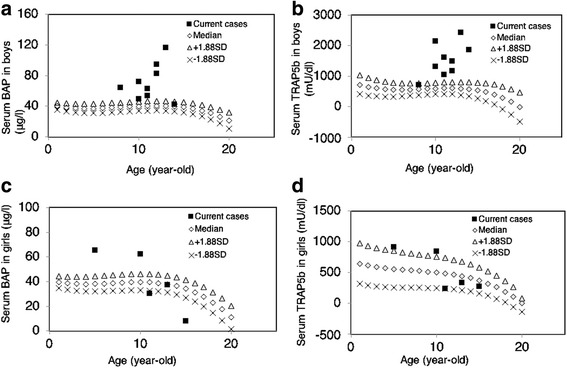
Distribution of bone metabolism markers in boys and girls according to age. Median and ±1.88SD values were referenced from a previous report [23]. (**a** and **b**) BAP and TRAP5b in boys with CNO were higher than that in the age-matched normal population, indicating higher bone metabolic turnover in boys with CNO. (**c** and **d**) BAP and TRAP5b in girls with CNO aged <10 years were higher than that in the age-matched normal population. These bone metabolic biomarkers were relatively lower in girls with CNO aged >10 years. BAP, bone alkaline phosphatase; TRAP5b, tartrate-resistant acid phosphatase serum band 5; CNO, chronic nonbacterial osteomyelitis

The BMD at L2-L4 was compared to that of a Japanese reference population, and the results were expressed as Z-scores. The mean Z-score at the lumbar spine in our patients with CNO was −1.7, and all of our patients had a BMD that was lower than the sex- and age-matched normal values (Fig. 1, range − 0.3 to −3.4). In boys, the BAP and TRAP5b values were higher than those in the age-matched normal population, indicating higher bone metabolic turnover (Fig. 2a, b). In contrast, the BAP and TRAP5b values in girls decreased from higher to lower with aging (Fig. 2c, d).

**Fig. 3:**
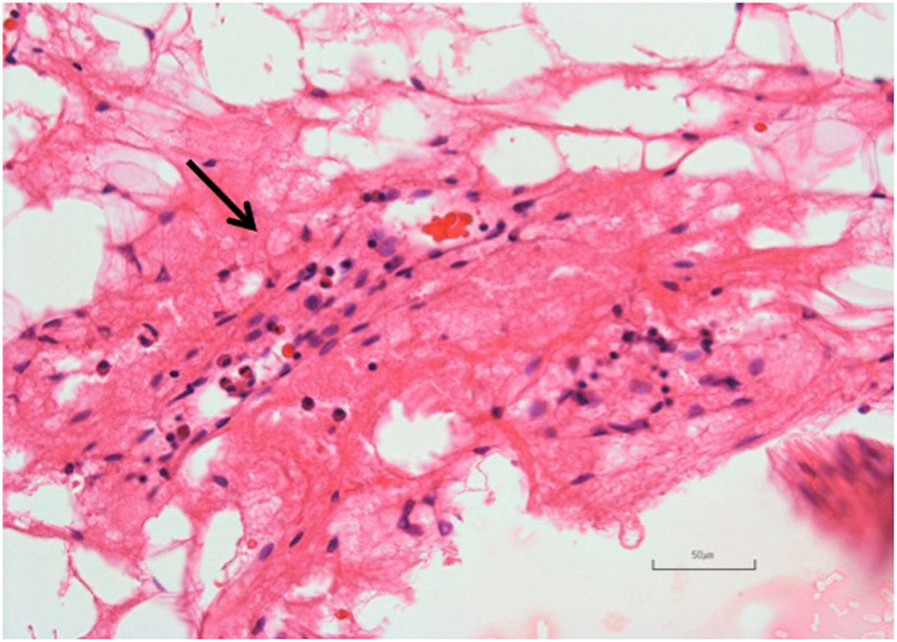
Photomicrograph of the bone biopsy specimen. *Black arrow* shows the region where increased lymphocytes, histiocytes, and plasma cells infiltrate the bone. Infiltration of neutrophils is not evident

Bone biopsies were performed for the confirmation of osteomyelitis, and for exclusion of bacterial infection and tumorous lesions in all patients. On histological analysis, increased infiltration of lymphocytes and plasma cells were observed in 11 cases; however, infiltration of neutrophils was not evident in all cases (Fig. 3). Microbiological cultures demonstrated no evidence of bacterial infection in all patients.

**Fig. 4:**
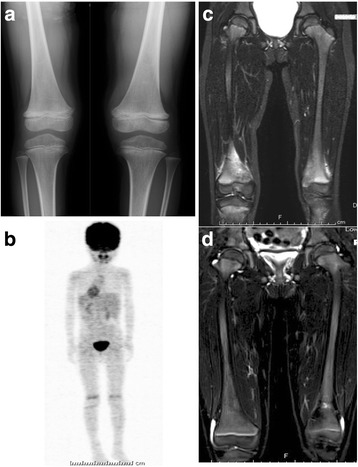
Representative case of a 5-year-old girl with CNO. The patient had a 3-month history of pain in both knees, and achieved clinical remission after the initial treatment. (**a**) Radiographic examination showed sclerosis in the distal metaphysis of both femurs. (**b**) FDG-PET image demonstrating increased multiple FDG uptakes in both distal femurs and right proximal tibia. (**c**) T2-STIR MRI scan at first presentation showing high signal in both distal femurs and right proximal tibia. (**d**) T2-STIR MRI scan at 6 months after the initiation of first-line treatment showing remission of high signals in both femurs and right tibia. CNO, chronic nonbacterial osteomyelitis; FDG-PET, fluorodeoxyglucose-positron emission tomography; T2-STIR MRI, T2-signal intensity in short tau inversion recovery magnetic resonance imaging

Five of the 14 patients were already administered antibiotics at previous hospitals before the diagnosis of CNO. Two patients were received short-term corticosteroid treatment. After presentation to our hospital, 11 patients (79%) were treated with NSAIDs in combination with a bisphosphonate and 3 patients (21%) were treated with NSAIDs alone (Table 1). The first-line therapy induced a partial response in 42% (6 of 14) and a clinical remission in 29% (4 of 14) during the 6-month treatment period. Clinical and imaging assessments revealed no response to the first-line therapy in four patients. A representative case of clinical remission is shown in Fig. 4. There was no difference in demographic data between group 1 patients with partial response or clinical remission, and group 2 patients with no response (Table 2). The BMD, SUV max, and serum data were also not significantly different between groups 1 and 2; however, serum IgG was significantly higher and the number of bone lesions detected on T2-STIR MRI was significantly greater in group 2 (Table 2).

**Table 2:** Demographic, serum inflammatory, and bone metabolic data and imaging findings

	group 1 (*n* = 10)	group 2 (*n* = 4)	*p* Value
Age (years)	10.3 (8.7–11.9)	12.7 (10.3–15.2)	0.09
Sex, female (%)	40	25	0.62
Time between first symptom to diagnosis (month)	8 (3.3–12.6)	9.2 (1.9–16.6)	0.76
CRP (mg/dL)	0.47 (0.01–1.23)	0.79 (0.01–1.79)	0.64
ESR (mm/h)	24.2 (6.6–41.8)	18.3 (9.6–46.1)	0.69
IgG (mg/dL)	943 (621–1266)	1607 (1097–2117)	<0.05
BAP (μg/L)	54.8 (36.3–73.4)	73.1 (43.8–102.4)	0.27
TRAP5b (mU/dL)	1205 (713–1696)	1095 (318–1871)	0.79
SUV max on FDG-PET	1.6 (1.1–4.1)	1.8 (1.1–4.1)	0.38
Number of bones with high accumulation on FDG-PET	4.2 (2.1–6.3)	6.3 (3.0–9.5)	0.27
Bone lesions with high signal on T2-STIR MRI	7.1 (1.2–13.0)	23.5 (14.2–32.8)	<0.01
Z score(−) of BMD	1.9 (1.2–2.6)	2.5 (1.3–3.7)	0.37

Group 1, patients with response to first-line treatment; group 2, patients with no response to first-line treatment. All data in groups 1 and 2 indicate mean values (range)

*Abbreviations*: *CRP* C-reactive protein, *ESR* erythrocyte sedimentation rate, *IgG* immunoglobulin G, *BAP* bone alkaline phosphatase, *TRAP5b* tartrate-resistant acid phosphatase serum; band 5, *SUV max*, maximum standardized uptake value, *FDG-PET* fluorodeoxyglucose-positron emission tomography, *T2-STIR MRI* T2-signal intensity in short tau inversion recovery magnetic resonance imaging, *BMD* bone mineral density

Second-line therapy was introduced to four patients in group 2. Methotrexate at 10 mg/m^2^ per week and infliximab at a dose of 5 mg/kg were administered (at 8-week intervals). After the induction of second-line therapy, all four patients were able to achieve a partial response.

## Discussion

The clinical features of CNO was first reported by Giedion et al. in 1972 [5]. CNO can be either acute or chronic, and unifocal or multifocal. Several studies have demonstrated the relations of immunological abnormality and gene mutations to CNO; however, the underlying cause has not yet been clarified [26]. Investigation of clinical features and laboratory tests cannot provide specific information about CNO, and the diversity of symptoms complicate the diagnosis [10, 11]. The diagnostic criteria have been established previously [4] but were not clinically useful [27]. Among the frequent pediatric complaints, it can be a challenge for the clinician to properly detect important symptoms that contribute to the earlier diagnosis of CNO. In the current study, we report the demographic, inflammatory, and bone metabolic features in 14 cases of CNO. The average age and sex ratio were similar to those previously reported in large cohort studies in Europe and northern Europe [24, 28]. The time required for the diagnosis in our patients represents the difficulty of the diagnosis of this disease. Inflammatory assessment by using CRP and ESR demonstrated a lack of systemic inflammation in some patients with CNO, which also complicates the diagnosis. Elevation of IgG may represent immunological involvement; however, IgG might not be specifically increased in CNO. As previously reported, detection of osteomyelitis on imaging modalities may have the best accuracy for diagnosis, and may exclude synovial inflammatory diseases, growing pains, and psychiatric problems [22]. Imaging modalities also play important roles in screening for multifocal inflammatory lesions, especially asymptomatic regions that sometimes result in irreversible bony change in CNO [1, 2].

The imaging modalities used in the diagnosis of CNO include plain radiography, MRI, and nuclear medicine scanning [14, 16]. Whole-body MRI (WB-MRI) has been used for the assessment of disease activity in children with CNO in several studies [1, 18, 29]. Voit et al. demonstrated the discrepancies between clinical symptoms and inflammatory lesions on WB-MRI in 17 cases of CRMO [1]. In their study, three CRMO cases with asymptomatic vertebral osteomyelitis resulted in vertebral body fracture in their long-term follow-up [1], indicating the importance of whole-body scanning for the screening of asymptomatic osteomyelitis. A case report introduced the potential utility of FDG-PET in detecting active bone lesions in CRMO [16]. In the current study, FDG-PET detected asymptomatic vertebral uptake regions in three cases. All three cases were treated with a bisphosphonate, and vertebral deformities were not found in any case during the follow-up period. The advantages of FDG-PET are it can quantify the severity of active inflammation through the measurement of the SUV [16] and facilitate the exclusion of inflammatory diseases or tumors. Although findings in FDG-PET did not show differences between responders and nonresponders to the first-line treatment in the current study, the numbers of bone lesions detected on MRI were significantly greater in nonresponders. This finding indicated that the number of inflammatory lesions may importantly affect the response to treatment, which agree with the results of previous large cohort studies [22, 24].

There is a consensus that NSAIDs are beneficial for patients with CNO and NSAIDs are the widely-used first-line treatment in this disorder [15]. NSAIDs provides pain reduction, however, it is not always equal to the remission of CNO [11]. Therefore, if NSAIDs treatment failed to control CNO, corticosteroids [6, 30], MTX, or anti-TNFα therapy [31, 32] are reportedly used as the second-line therapy. Several pediatric reports have also suggested the efficacy of bisphosphonates for symptomatic improvement in CNO [17, 33–36]. Our data on bone metabolism demonstrated that patients with CNO have lower BMD in the lumbar spine than age-matched controls. Elevation of bone formation and resorption biomarkers in boys and younger girls with CNO indicated a high turnover of bone metabolism, which may be responsible for the osteoporotic change in BMD. Considering previous reports with histological evidence of increased osteoclasts in CNO [37], increase in bone resorption may initiate abnormal bone turnover that secondarily increases bone formation compared with the age-specific norms in our patients. As shown in a previous study, asymptomatic vertebral osteomyelitis can cause vertebral-body fracture [1]. Because bisphosphonates are powerful inhibitors of osteoclastic bone resorption and reduce excessive bone turnover that may contribute anti-inflammatory, pain-modifying, and bone deformity-preventive effects [17, 18, 38], bisphosphonate treatment is a logical therapy for CNO.

Evaluating the treatment responses in patients with CNO is difficult because the variety of recurrence patterns precludes simple assessment. The clinical features and outcomes in CNO are variable, which complicates the identification of patients who require intensive treatment to control their severe disease activity. What triggers the initial episode is unknown; however, specific biomarkers will provide important information for selecting patients with a higher risk for prolonged disease courses. In the current study, prolonged active inflammation severely impaired daily activity in 4 of 14 patients with CNO. Similarly to MRI, as described above, measurement of IgG, which was highly produced in patients with no response to the first-line therapy in our study, may facilitate the prediction of the chronicity of CNO. A recent study demonstrated TNF-α as an increased cytokine in CRMO [39]. Therefore, the combination of methotrexate and TNF blockers seem to be a next option if first-line treatment is unsuccessful [31]. This aggressive therapy ensures better results in a proportion of patients with CNO.

Our study had several limitations. First, we enrolled only 14 patients. In our small study population, higher serum IgG levels and greater numbers of inflammatory lesions likely have an association with prolonged disease activity in CNO. For the accurate determination of factors for disease prognosis, multivariable analysis on a larger number of patients will be required. However, despite the small number of patients, our data in the current study clearly demonstrated the involvement of abnormal bone turnover. Moreover, the presence of asymptomatic inflammatory regions was demonstrated on FDG-PET in 3 of 14 patients, indicating the importance of whole-body scanning. Second limitation of our study is radiation exposure and limited versatility of FDG-PET. FDG-PET provides valuable information about inflammatory regions that may not be easily detected by other diagnostic imaging modalities, although FDG-PET is available in limited facilities and exposes higher radiation than other imaging modalities. Because the WB-MRI protocol involves obtaining STIR images alone to keep the imaging duration within acceptable limits for children [1, 13], we used FDG-PET as a whole-body scanning and MRI were conducted only on the locations of clinical symptoms or uptake regions detected on FDG-PET for the focal assessment of bone abnormalities. Since we did not conduct WB-MRI, we could not compare systemic uptakes of FDG-PET with abnormal findings of MRI in the current study. As our study supports the usefulness of MRI in CNO, the establishment of a protocol for the optimal setting of WB-MRI that can be performed within an acceptable scanning time frame remains a future goal. Third limitation of our study is a short follow-up period. Since there is significant overlap between CNO and IBD, psoriasis or ankylosing spondylitis, we need longer follow-up of our patients to see if they will develop gastrointestinal symptoms or rash to elucidate the relevance of other autoimmune disorders with CNO.

Further study with more patients will be required to determine the factors predicting intractable CNO, and to suggest the exact treatment strategy for preventing prolonged symptoms and disruption of daily activity in patients with CNO.

## Conclusion

Results of our study indicated the involvement of systemic abnormal bone turnover in characteristic of CNO. Whole-body scanning was useful for detection of multiple inflammatory focus including asymptomatic regions. Higher serum IgG levels and greater numbers of inflammatory lesions in MRI associated with prolonged disease activity in CNO patients.

## Abbreviations

BAP: Bone alkaline phosphatase
BMD: Bone mineral density
CNO: Chronic nonbacterial osteomyelitis
CRMO: Chronic recurrent multifocal osteomyelitis
CRP: C﻿-reactive protein
ESR: Erythrocyte sedimentation rate
FDG-PET: Fluorodeoxyglucose-positron emission tomography
IgG: Immunoglobulin G
MRI: Magnetic resonance imaging
SAA: Serum amyloid A
SUV: Standardized uptake value
T2-STIR: T2-signal intensity in short tau inversion recovery
TRAP5b: Tartrate-resistant acid phosphatase serum band 5
WB: Whole-body
